# Ablation of TRPV1+ Afferent Terminals by Capsaicin Mediates Long-Lasting Analgesia for Trigeminal Neuropathic Pain

**DOI:** 10.1523/ENEURO.0118-20.2020

**Published:** 2020-05-29

**Authors:** Sheng Wang, Chao Bian, Jiale Yang, Vipin Arora, Yiwei Gao, Feng Wei, Man-Kyo Chung

**Affiliations:** Department of Neural and Pain Sciences, School of Dentistry, University of Maryland, Program in Neuroscience, Center to Advance Chronic Pain Research, Baltimore, MD 21201

**Keywords:** analgesia, capsaicin, neuropathic pain, orofacial pain, TRPV1

## Abstract

Trigeminal neuropathic pain (TNP) is often resistant to current pharmacotherapy, and there is a pressing need to develop more efficacious treatments. Capsaicin is a pungent ingredient of chili peppers and specifically activates transient receptor potential vanilloid subtype 1 (TRPV1), a Ca^2+^-permeable ion channel. Topical capsaicin invariably induces burning pain. Paradoxically, the transient pain is often followed by prolonged attenuation of the preexisting pathologic pain from the same region. However, the mechanisms underlying capsaicin-induced analgesia are not well understood. Although the reports of the involvement of TRPV1 and TRPV1+ afferents in neuropathic pain are controversial, we recently demonstrated that TRPV1 and TRPV1+ afferents are involved in mechanical hyperalgesia in mice with chronic constriction injury of the infraorbital nerve (ION-CCI). Consistently, chemogenetic inhibition of TRPV1-lineage (TRPV1-LN) afferents attenuated mechanical hyperalgesia and ongoing pain. In mice with ION-CCI, we found that a single focal injection of capsaicin into facial skin led to attenuation of mechanical hyperalgesia over two weeks. Capsaicin treatment also attenuated secondary hyperalgesia in extraterritorial mandibular skin. Furthermore, capsaicin treatment decreased ongoing pain. Longitudinal *in vivo* two-photon imaging of cutaneous nerve fibers showed that such capsaicin-induced analgesia is correlated with cutaneous nerve terminal density. Furthermore, preventing capsaicin-induced ablation of afferent terminals by co-administration of capsaicin with MDL28170, an inhibitor of calpain, abolished capsaicin-induced analgesia. These results suggest that a single focal injection of capsaicin induces long-lasting analgesia for neuropathic pain via selective ablation of TRPV1+ afferent terminals and that TRPV1+ afferents contribute to the maintenance of TNP.

## Significance Statement

Capsaicin has long been used as an analgesic to treat chronic pain conditions. Topical capsaicin is an FDA-approved treatment for neuropathic pain. However, the mechanisms underlying capsaicin-induced analgesia have been enigmatic for centuries. Despite evidence for clinical analgesia, data supporting the analgesic effects of capsaicin on neuropathic pain in preclinical model is rare. We found that a single focal injection of capsaicin to facial skin robustly attenuated trigeminal neuropathic pain (TNP) in mice for longer than two weeks, which is mediated by localized ablation of transient receptor potential vanilloid subtype 1 (TRPV1)+ terminals in the skin. These results suggest that TRPV1+ afferents contribute to the maintenance of TNP and that capsaicin injection could be a safe and effective treatment for TNP.

## Introduction

Trigeminal nerve damage due to accidents, orthognathic surgery, or dental procedures often causes trigeminal neuropathic pain (TNP), which becomes chronic, lasting more than a year, in one third of patients (Agbaje et al., 2016). Since TNP responds more poorly to conventional medications than does spinal traumatic neuropathic pain (McQuay et al., 1996; Haviv et al., 2014), development of novel methods to manage TNP is crucial.

Transient receptor potential vanilloid subtype 1 (TRPV1) is a pro-nociceptive ion channel protein activated by capsaicin, heat and multiple endogenous ligands (Chung et al., 2011). Interestingly, administration of capsaicin not only produces transient burning pain but also induces analgesia for neuropathic pain conditions (Szallasi and Blumberg, 1999; Chung and Campbell, 2016). Topical capsaicin relieves self-reported pain ratings by >30% for approximately three months in ∼40–45% of postherpetic neuralgia patients (Mou et al., 2013; Treede et al., 2013). Topical capsaicin not only attenuates pain aversion but is also reported to attenuate mechanical hyperalgesia in patients with peripheral neuropathy from multiple etiologies (Kennedy et al., 2010; Zis et al., 2014). The therapeutic effects of topical capsaicin on dynamic allodynia are even greater than those of pregabalin (Cruccu et al., 2018). Thus, targeting TRPV1+ afferents using topical capsaicin is a promising approach to treating neuropathic pain, especially in patients whose hyperalgesia is likely maintained by sensitized nociceptors (Baron et al., 2017). In addition to topical application, intraarticular or intrathecal injection of capsaicin or resiniferatoxin (RTX), a potent TRPV1 agonist, is also under active development for treating chronic pain (Iadarola et al., 2018; Sapio et al., 2018; Stevens et al., 2019).

Despite clear therapeutic benefits of capsaicin, roles of TRPV1 and TRPV1+ nociceptors in neuropathic pain are controversial, and therefore, the logical rationale for capsaicin therapy is not well supported by mechanistic insights. A majority of reports dispute the involvement of these entities in neuropathic mechanical allodynia: knock-out or knock-down of TRPV1 does not affect mechanical hyperalgesia following peripheral neuropathy in mice (Caterina et al., 2000; Hirai et al., 2014). Genetic ablation of TRPV1+ neurons or chemical ablation or desensitization of TRPV1+ afferents neither prevented nor attenuated mechanical hyperalgesia following neuropathy (Ossipov et al., 1999; King et al., 2011; Mishra et al., 2011; Abooj et al., 2016). These data contrast sharply with the aforementioned clinical data, as well as with multiple preclinical studies implicating TRPV1 and TRPV1+ afferents in mechanical hyperalgesia following neuropathy (Pomonis et al., 2003; Rashid et al., 2003; Kanai et al., 2005; Christoph et al., 2007; Watabiki et al., 2011). Our recent study showed that TRPV1 and TRPV1+ afferents are clearly involved in orofacial mechanical hyperalgesia following chronic constriction injury of infraorbital nerve (ION-CCI) in mice (Kim et al., 2014). Injection of TRPV1 antagonist into the trigeminal subnucleus caudalis (Vc) effectively attenuated mechanical hyperalgesia, while systemic treatment with RTX prevented development of mechanical hyperalgesia following ION-CCI. However, it is not known whether peripheral administration of capsaicin reduces trigeminal hyperalgesia in animals with ION-CCI.

Capsaicin-induced long-lasting analgesia in patients might occur through multiple mechanisms (Chung and Campbell, 2016). A dominant presumption is that analgesia produced by local administration of capsaicin is associated with the localized ablation of TRPV1+ peripheral fibers based on the observations that topical or intradermal injection of capsaicin denervates intraepidermal nerve fibers in humans (Simone et al., 1998; Malmberg et al., 2004; Anand and Bley, 2011). However, causal contribution of capsaicin-induced ablation of TRPV1+ nerve terminals to the analgesic effects on neuropathic pain has not been investigated. It appears to be straightforward that ablation of peripheral nociceptive fibers produces analgesia. However, considering the fact that multiple neuropathic pain conditions are accompanied by reductions in intraepidermal nerve fiber density (Landowski et al., 2016), it is important to determine the causal role for capsaicin-induced ablation of TRPV1+ fibers in capsaicin-induced analgesic effects. We recently showed that capsaicin ablates TRPV1+ nerve terminals through TRPV1-mediated Ca^2+^ influx followed by the activation of calpains, which are Ca^2+^-dependent proteases (Wang et al., 2017b). MDL28170, an inhibitor of calpains, reduced capsaicin-induced ablation of TRPV1+ afferent terminals *in vitro* and *in vivo*. Importantly, MDL28170 prevented capsaicin-induced thermal hypoalgesia in hindpaw (Wang et al., 2017b). However, it is not known whether such capsaicin-induced calpain-dependent ablation of TRPV1+ nerve terminals is necessary for analgesia of neuropathic pain conditions.

In this study, we tested the efficacy of focal injection of capsaicin in a mouse model of TNP and tested the hypothesis that TRPV1+ afferents contribute to the maintenance of TNP and capsaicin-induced ablation of peripheral nociceptive fibers is necessary for capsaicin-induced long-lasting analgesia.

## Materials and Methods

### Animals

C57bl/6J (Jax #00064), TRPV1^Cre^ (Jax #017769; Cavanaugh et al., 2011), R26^LSL-tdTomato^ (Jax #007914), R26^mT/mG^ (Jax #007576), and R26^LSL-hM4Di^ (Jax #026219) mice were purchased from The Jackson Laboratory. TRPV1^Cre^ mice (Cavanaugh et al., 2011) express Cre recombinase under the control of TRPV1 promoter and, when crossed with a Cre-dependent reporter line, show labeling of afferents that have expressed TRPV1 at any stage of development (TRPV1-lineage; TRPV1-LN). Of these TRPV1-LN neurons, about half express TRPV1 in adult sensory ganglia (Cavanaugh et al., 2011). We generated a mouse line expressing tdTomato in TRPV1-LN neurons (TRPV1^tdTomato^) by crossing TRPV1^Cre^ mice and R26^LSL-tdTomato^ mice. A mouse line expressing membrane-bound GFP (mGFP) from TRPV1 locus (TRPV1^mGFP^) was generated by crossing TRPV1^Cre^ mice and R26^mT/mG^ mice. A mouse line expressing inhibitory designer receptor exclusively activated by designer drugs (DREADD) under control of the TRPV1 promoter (TRPV1^hM4Di^) was generated by crossing TRPV1^Cre^ mice and R26^LSL-hM4Di^ mice. All procedures were conducted in accordance with the National Institutes of Health *Guide for the Care and Use of Laboratory Animals* and were performed under protocols approved by The University of Maryland Animal Care and Use Committees. Adult (eight-week-old) male and female mice were used for the experiments. Animals were group-housed under standard conditions with *ad libitum* access to water and food.

### TNP model

To produce the TNP model, we performed chronic constriction injury of the infraorbital nerve (ION-CCI; Kim et al., 2014). Loose ligatures of the unilateral infraorbital nerve were done via an intraoral approach to keep orofacial skin intact for measurement of mechanical sensitivity. Mice were anesthetized with intraperitoneal injection of mixed ketamine (100 mg/kg) and xylazine (10 mg/kg). The head of the mouse was positioned in lateral recumbency and four limbs fixed on the table with adhesive tape. A 5- to 7-mm-long incision was made along the gingivobuccal margin in the buccal mucosa, beginning immediately next to the first molar. The ION was freed from surrounding connective tissues by a glass rod and clearly visualized using a surgical microscope. At 3–4 mm from the nerve where its branches emerge from the infraorbital fissure, the ION was loosely tied with two chromic gut (4.0) ligatures, 2 mm apart. The incision was closed using veterinary tissue glue. The sham-operated mice received only a unilateral nerve exposure without ligatures.

### Behavioral assay for mechanical sensitivity

All behavioral tests were conducted under blind conditions. To reduce any effects of restraint, the mouse was habituated for 20 min/d for 3 d to stand on or lean against a regular leather work hand glove worn by the experimenter. As previously described (Kim et al., 2014), a series of calibrated von Frey (VF) filaments with bending forces ranging from 0.008 to 4 g were applied to the orofacial skin. For assessing primary or secondary hyperalgesia, these filaments were applied to the skin just behind the vibrissa pad within the infraorbital territory (V2) and the skin just below the ear within the mandibular nerve territory (V3), respectively. A brisk or active withdrawal of the head from the probing filament was defined as a nocifensive response. Each VF filament was applied five times at intervals of a few seconds. The response frequencies [(number of responses/number of stimuli) × 100%] to a range of VF filament forces were determined and S-R curves were plotted. Mechanical threshold was measured as the lowest intensity at which animals escaped from the stimuli over 50% of chance. In this mouse ION-CCI model, stable and long-lasting mechanical hyperalgesia and allodynia develops in maxillary V2 skin as well as in extraterritorial mandibular V3 skin within one week and persists over three months (Kim et al., 2014).

### Capsaicin injection into facial skin

To determine the effects of focal capsaicin on mechanical hyperalgesia, capsaicin was injected at day 14 following ION-CCI. A single bolus of either vehicle (20 µl, 25% PEG300 in water) or capsaicin (10 µg in 20 µl of vehicle) was subcutaneously injected into the skin just behind the vibrissa pad, in which VF testing was performed for primary hyperalgesia. VF testing was performed up to four weeks except in a cohort that was tested up to nine weeks for determining the effects of two times of capsaicin injection.

### Intra-Vc microinjection of drug

Microinjection into the Vc was performed as described previously (Kim et al., 2014; Wang et al., 2017a). Animals were anesthetized by intraperitoneal injection of ketamine and xylazine cocktail and placed in a Kopf stereotaxic apparatus. After a midline incision, an opening was made in the skull. The needle of a 0.5-μl Hamilton microsyringe was placed in the left or right Vc regions according to the stereotaxic coordinates of the mouse brain (7.80 mm anterior and 4.3 mm ventral to bregma and ±1.60 mm lateral to the midline; Paxinos and Franklin, 2008). Clozapine N-oxide (CNO; 0.1 µg/1 µl) or 2% lidocaine (0.5 μl) was administered for 1 min. Following injection, the needle was held in the tissue for 2 min to allow diffusion before removal.

### Conditioned place preference (CPP) assay

To test effects of focal capsaicin treatment on CCI-induced pain aversion, conditional preference place (CPP) was performed as previously described (Wang et al., 2018). Mice with ION-CCI or sham surgery were conditioned by intra-Vc injection of 2% lidocaine or vehicle with capsaicin or saline pretreatment. In another experiment using TRPV1^hM4Di^ and TRPV1^Cre^ mice, CNO or vehicle was injected for conditioning. The CPP apparatus consisted of a rectangular chamber with one side compartment measuring 23 × 26 cm with black walls and grids on the floor, a central compartment measuring 23 × 11 cm with clear Plexiglas walls and a Plexiglas floor, and another side compartment measuring 23 × 26 cm with white walls and a mesh metal floor. Mouse position during each day of testing was monitored. All mice were exposed to the environment with full access to all chambers for 30 min each day for days 1–3. On day 3, behavior was recorded for 15 min and analyzed to verify absence of preconditioning chamber preference. Following the preconditioning phase, mice underwent conditioning for 1-d trial with alternate treatment-chamber pairings at day 4. Mice received vehicle and paired with a chamber in the morning. In the afternoon, drugs such as lidocaine or CNO were administered and paired with the other chamber. Mice were placed in the paired chamber with no access to the other chamber at 30 min after intra-Vc injection of vehicle or drugs, by which time animals had completely recovered from anesthesia by isoflurane. Drug and chamber pairing were counterbalanced. On day 5, mice were placed into the central chamber with access to all chambers. For 15 min, with the mouse in a drug-free state, time spent in each of the chambers was recorded. Increased time spent in a chamber indicates preference for that chamber.

### Fluorescence imaging of cutaneous afferents *in vivo*

Imaging was performed in the dark without any stimuli. After anesthetized with ketamine (100 mg/kg, i.p.) and xylazine (10 mg/kg, i.p.), adult TRPV1^tdTomato^ or TRPV1^mGFP^ mice were placed prone on the animal platform with an electrical heating pad automatically around 38°C. A hindpaw was extended and mounted on the platform. The glabrous flat skin was located at the mid plantar region behind the footpad between the secondary and third digits of the hindpaw. Saline was dropped on the mid plantar region and covered with a coverslip, and then a water immersion fluorescence objective was lowered into distilled water on the coverslip. TRPV1-LN afferent terminals expressing tdTomato or mGFP within the epidermis and dermis were imaged using a two-photon microscope (Scientifica) with a Ti:Sapphire laser (Mai Tai; Spectra Physics) using a Nikon X16 water-immersion lens (0.8 NA; 3.0 mm WD). The laser wavelength for two-photon excitation was 1040 nm for tdTomato and 900 nm for GFP, and the laser power was maintained at ≤25 mW. Consecutive z-stack images were captured at 2-μm (TRPV1^tdTomato^ mice) or 0.5-μm (TRPV1^mGFP^ mice) depth intervals from the selected skin spots by using photomultiplier tubes in whole-field detection mode until the fluorescence signal became undetectable. *XZ*-axis orthogonal view of the skin was reconstructed using “volume viewer” plugins in ImageJ (NIH). The raw images were processed to remove the nonspecific autofluorescence in the skin. To simplify the measurement procedure, the *Z* projection was applied to a stack of all imaging sections and a single merged imaging of the skin spot was created. With ImagingJ analysis, a threshold was set to completely eliminate the background grayscale and binary images were generated. GFP+ area and entire region of interest (ROI) area were measured, and the ratio of the GFP+ area/ROI area was calculated.

### Drugs

Facial skin was injected with either vehicle (20 µl, 25% PEG300, and 75% H_2_O) or capsaicin (10 µg in 20 µl of vehicle). Pharmaceutical grade of capsaicin dissolved in PEG300 was kindly donated by the Centrexion Therapeutics Corporation. MDL28170 was from Tocris or Sigma. All other chemicals and drugs were purchased from Sigma-Aldrich. To determine the role of calpain, we injected 20 µl of 10 mm MDL28170 (200 nmol), which effectively prevented the ablation of TRPV1+ afferents in hindpaw (Wang et al., 2017b).

### Design and statistical analysis

The method of statistical analysis used in each dataset is indicated in the figure legends. Data from two groups were compared using Student’s *t* test. Data from three or more groups were compared using one-way ANOVA followed by Bonferroni *post hoc* test. The effects of pharmacological manipulations at different time points were analyzed with two-way ANOVA with repeated measures (RM). All multiple group comparisons were performed by Bonferroni *post hoc* test. Data are presented as mean ± SEM. The criterion for statistical significance was *p* < 0.05. All statistical analyses were performed using GraphPad Prism 6.0.

## Results

### Chemogenetic inhibition of TRPV1-LN afferents attenuates TNP in mice with ION-CCI

**Figure 1.**
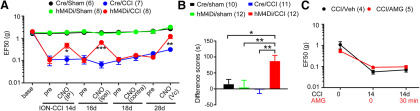
Chemogenetic inhibition of TRPV1+ afferent terminals attenuates mechanical hyperalgesia and ongoing pain in mice with ION-CCI. ***A***, Effects of CNO on mechanical hyperalgesia following ION-CCI in mice expressing inhibitory DREADD in TRPV1-LN neurons (TRPV1^Cre^;R26^LSL-hM4Di^; hM4Di) or TRPV1^Cre^ mice (Cre). CNO was repeatedly injected intraperitoneally (40 µg/20 µl), to ipsilateral (ipsi; 20 µg/10 µl) or contralateral (contra; 20 µg/10 µl) V2 skin or intra-Vc (Vc; 0.1 µg/1 µl) at indicated time points. Mechanical sensitivity is presented as EF50 (the mechanical force that produced a 50% response frequency) in the ipsilateral V2 skin; *p < 0.05, **p < 0.01, ***p < 0.001 in Bonferroni *post hoc* test following two-way ANOVA. Throughout the figures, numbers in parenthesis represent the number of mice in each group. ***B***, Preconditioning habituation started at four to five weeks after CCI or sham surgery. Following the habituation period, the mice received intra-Vc microinjection of vehicle (0.5-µl saline) in the ipsilateral side and paired with a chamber for 30 min. Four hours later, the same mouse received 0.5-µl CNO paired with the other conditioning chamber for 30 min. On the following test day, the mice were placed in the CPP chambers with free access to all chambers to measure time spent in both chambers. Mice were recorded for 15 min, and difference scores were calculated by subtracting time spent in CNO-paired chambers before conditioning from the time after conditioning; *p < 0.05, **p < 0.01 in Bonferroni *post hoc* test following two-way ANOVA. ***C***, The effects of AMG9810 (100 nmol/10 µl), a TRPV1 antagonist, or vehicle injected into the ipsilateral V2 skin on mechanical sensitivity in the ipsilateral V2 skin of mice with ION-CCI.

Our previous study suggested that TRPV1 in the central terminals of primary afferents in the Vc and TRPV1+ afferents mediates orofacial mechanical hyperalgesia following ION-CCI (Kim et al., 2014). To further determine the role of TRPV1+ afferents in mechanical hyperalgesia in mice with ION-CCI, we performed the chemogenetic inhibition using TRPV1^hM4Di^ mice, which was produced by crossing TRPV1^Cre^ with Rosa26^LSL-hM4Di^ mice (Fig. 1*A*). Basal mechanical sensitivity and ION-CCI-induced mechanical hyperalgesia were comparable in TRPV1^hM4Di^ and TRPV1^Cre^ animals. However, mechanical hyperalgesia was significantly suppressed by intraperitoneal administration of CNO in TRPV1^hM4Di^, an effect fully reversible after 1 d. To examine whether functional blockade of TRPV1-LN peripheral nerve terminals mediates CNO-induced analgesia, we locally injected CNO into the ipsilateral facial skin in the same animals, and found that CNO also significantly attenuated mechanical hyperalgesia. In contrast, the contralateral injection of CNO did not affect mechanical hyperalgesia, suggesting that the action of CNO was local and peripherally confined. Intra-Vc injection of CNO also significantly attenuated mechanical hyperalgesia, which supports our previous observation that central terminals of TRPV1+ afferent are involved in mechanical hyperalgesia (Kim et al., 2014). Administration of CNO via any route did not affect mechanical sensitivity in sham groups either in TRPV1^hM4Di^ or TRPV1^Cre^ mice (data not shown). These results provide evidence that TRPV1-LN afferents contribute to mechanical hyperalgesia following ION-CCI. Moreover, silencing central terminals of TRPV1-LN afferents within Vc promoted CPP in TRPV1^hM4Di^ mice with ION-CCI but not in TRPV1^Cre^ mice (*F*_(3,41)_ = 5.62, *p* = 0.002; Fig. 1*B*), which further supports the hypothesis that TRPV1+ afferents contribute to the maintenance of TNP.

We previously showed that pharmacological inhibition of TRPV1 in central terminals within the Vc attenuates mechanical hyperalgesia in mice with ION-CCI (Kim et al., 2014). However, injection of AMG9810, an antagonist of TRPV1, into ipsilateral skin did not affect mechanical hyperalgesia (Fig. 1*C*), suggesting that TRPV1 plays different roles at peripheral and central terminals. Together, these results indicate that peripheral TRPV1+ afferents but not TRPV1 channels expressed in its peripheral terminals contribute to the maintenance of mechanical hyperalgesia after trigeminal nerve injury.

### Capsaicin-induced long-lasting analgesia in mice with CCI-ION

**Figure 2.**
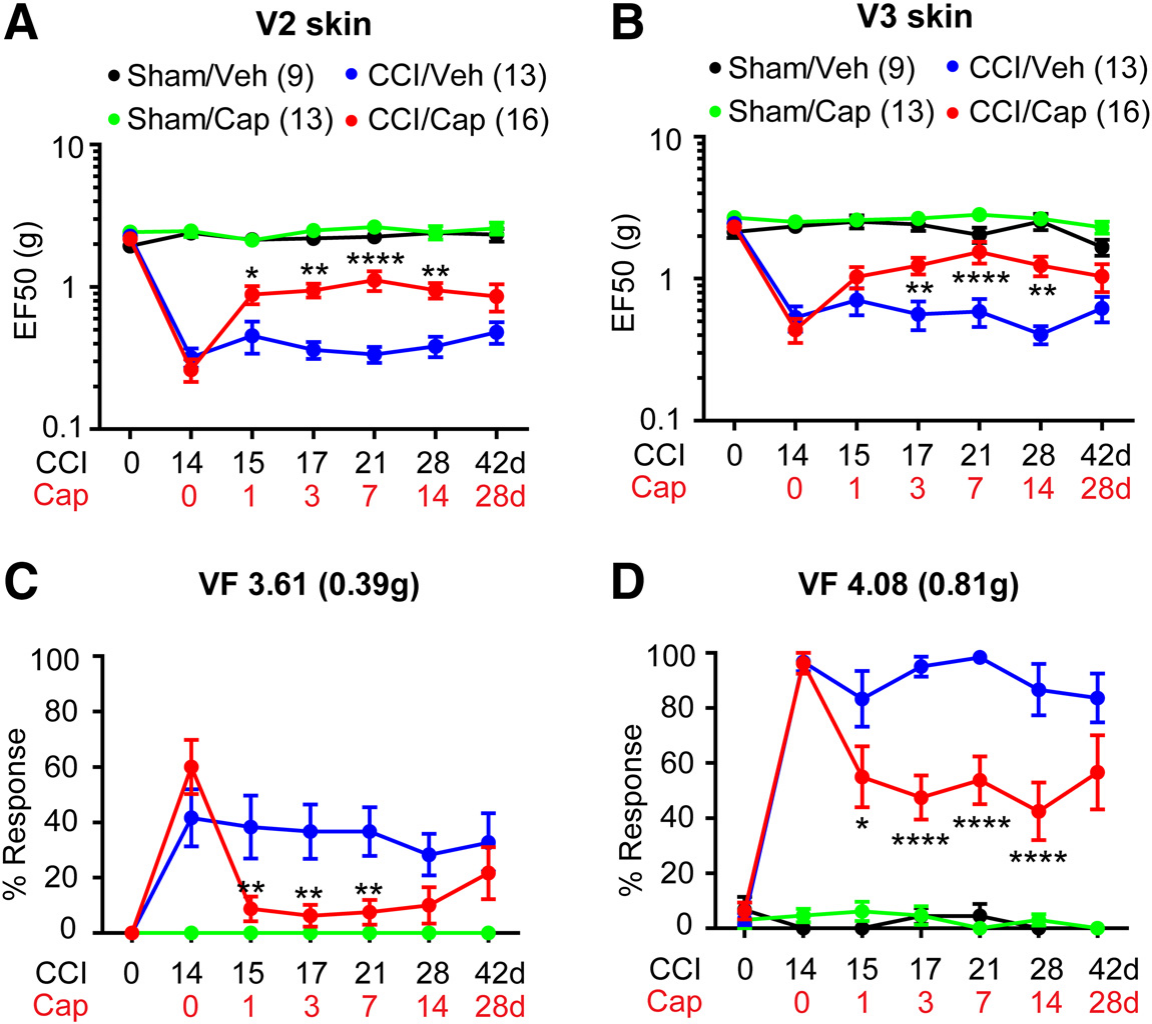
Single subcutaneous injection of capsaicin attenuates mechanical hyperalgesia in mice with ION-CCI. ***A***, ***B***, Mechanical sensitivity in the ipsilateral V2 skin (***A***) or in the ipsilateral V3 skin (***B***). Vehicle or capsaicin (10 µg) was subcutaneously injected into the ipsilateral V2 skin area 14 d after CCI or sham surgery. ***C***, ***D***, Percent response frequency in response to VF hair 3.61 (***C***) or 4.08 (***D***). Symbols and n numbers within parenthesis in ***A***, ***B*** are also applied to ***C***, ***D***. Two-way ANOVA followed by Bonferroni *post hoc* test (CCI/Veh vs CCI/Cap); *p < 0.05, **p < 0.01, ****p < 0.0001.

To further test the hypothesis that capsaicin-induced ablation of TRPV1+ peripheral terminals produces analgesia, we conducted focal application of capsaicin to determine the effects of specific targeting TRPV1+ afferents. After the full establishment of mechanical hyperalgesia two weeks after the unilateral ION-CCI, we injected capsaicin (10 µg) into maxillary V2 skin by subcutaneous injection under anesthesia. Then, mechanical sensitivity was tested at indicated time points after injection. The mice with ION-CCI exhibited a significant increase in mechanical threshold in V2 skin from 1 d (Fig. 2*A*). Capsaicin injection did not produce any significant changes of the mechanical threshold in the sham group. However, a single injection of capsaicin in mice with ION-CCI resulted in prolonged elevation of mechanical threshold from 1 d through 14 d compared with the vehicle group (drug, *F*_(1,151)_ = 45.47, *p* < 0.0001; time, *F*_(5,151)_, *p* = 0.0021; interaction, *F*_(5,151)_ = 3.15, *p* = 0.0099; two-way RM ANOVA). At 28 d, mechanical threshold was still elevated compared with the vehicle group but did not reach statistical significance. Capsaicin injection did not affect mechanical sensitivity of ipsilateral skin in the sham group. We further examined whether capsaicin treatment in V2 skin also changes hypersensitivity in V3 skin. Interestingly, we found that capsaicin injection confined only to V2 skin also attenuated mechanical hyperalgesia in V3 skin (Fig. 2*B*). When percent responses to two VF filaments were compared (Fig. 2*C*,*D*), the effects of capsaicin were more pronounced. VF filaments exerting 0.39 g produced withdrawal responses ∼60% of the time whereas VF filaments exerting 0.81 g did so almost 100% time in ION-CCI group. After injection of capsaicin, there were substantially no responses to VF-0.39 g, whereas responses to VF-0.81 g only occurred ∼50% of the time. ION-CCI did not produce mechanical hyperalgesia in the contralateral skin, and capsaicin injection into the ipsilateral skin did not affect mechanical sensitivity in the contralateral skin (data not shown).

### Capsaicin injection attenuates pain aversion in mice with CCI-ION

**Figure 3.**
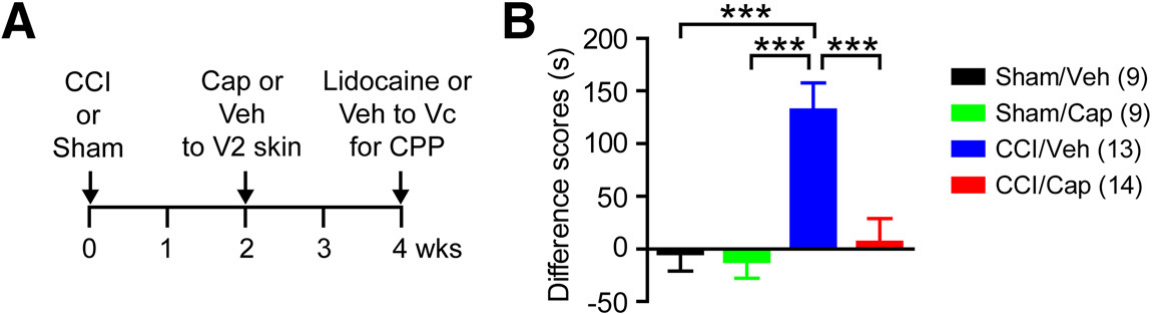
CPP test following capsaicin or vehicle injection in mice with CCI-ION or sham surgery. ***A***, Time course of CPP experiment. Preconditioning habituation started at four weeks after CCI or sham surgery (two weeks after cap or vehicle injection). Time spent in two chambers were recorded for 15 min (preconditioning trial). Following the habituation period, the mice received intra-Vc microinjection of vehicle (0.5-µl saline) in the ipsilateral side and paired with a chamber for 30 min. Four hours later, the same mouse received 0.5-µl 2% lidocaine paired with the other conditioning chamber for 30 min. On the following test day, the mice were placed in the CPP chambers with free access to all chambers to measure time spent in both chambers (testing trial). Mice were recorded for 15 min, and chamber preference in comparison with preconditioning time was analyzed. ***B***, Difference scores were calculated by subtracting time spent in lidocaine-paired chambers during preconditioning trial from the time during testing trial; ***p < 0.001 in Bonferroni *post hoc* test following one-way ANOVA.

We next evaluated the effect of capsaicin on affective or aversive pain using a CPP paradigm (Fig. 3*A*). At two weeks after ION-CCI or sham surgery, mice received vehicle or capsaicin injection into the ipsilateral V2 skin. Two weeks after capsaicin or vehicle injection, we conditioned the mice using intra-Vc injection of lidocaine or vehicle. Comparing time spent in the lidocaine-paired chamber, there was a significant interaction between time and treatment groups (*F*_(3,41)_ = 9.76, *p* < 0.0001; two-way RM ANOVA). Mice with ION-CCI receiving vehicle to V2 skin (CCI/Veh) spent a significantly longer time in the lidocaine-paired chamber on the testing day than on the preconditioning day, indicating analgesic effects of intra-Vc lidocaine on ongoing pain (preconditioning, 331 ± 27 s; testing, 462 ± 34 s; *n* = 13; *p* < 0.0001 in Bonferroni *post hoc* test following two-way ANOVA). In contrast, ION-CCI mice with capsaicin injection (CCI/Cap) spent almost equal amounts of time in lidocaine-paired chambers before and after the conditioning, indicating a lack of analgesia induced by lidocaine (preconditioning, 336 ± 39 s; testing, 343 ± 36 s; *n* = 14; *p* > 0.99). When difference scores were calculated (Fig. 3*B*), the CCI/Veh group showed significantly greater scores than sham groups and CCI/Cap groups (*F*_(3,41)_ = 9.76, *p* < 0.0001 in one-way ANOVA). The CCI/Cap group did not show a significant difference compared with sham groups. Overall, these results provide evidence that focally injected capsaicin leads to attenuation of ongoing affective pain.

### Capsaicin-induced ablation of TRPV1+ peripheral terminals correlates with analgesia

**Figure 4.**
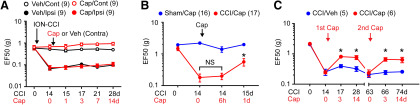
Properties of capsaicin-induced analgesia. ***A***, In mice with ION-CCI on the left side, the effects of capsaicin (red) or vehicle (black) injection into V2 skin on the right contralateral side. Mechanical sensitivity was measured from V2 skin in the contralateral side (open circles; capsaicin-injected right side) and ipsilateral side (filled circles; left side with ION-CCI). ***B***, The effects of capsaicin injection into the ipsilateral V2 skin on mechanical sensitivity in the ipsilateral V2 skin of mice with ION-CCI (red) or sham (blue) at indicated time points. Capsaicin-induced analgesia was not initiated at 6 h following capsaicin injection. One-way RM ANOVA followed by Sidak multiple comparison test; *p < 0.05 (vs Cap 0 d in CCI/Cap group), NS, not significant. ***C***, The effects of repeated injection of capsaicin into the ipsilateral V2 skin on mechanical sensitivity in the ipsilateral V2 skin of mice with ION-CCI at indicated time points. Analgesia by first capsaicin disappeared after seven weeks following injection. Second capsaicin produced analgesia again; *p < 0.05 (vs CCI/Veh group in each time point) in *post hoc* test following two-way RM ANOVA.

Injection of capsaicin into V2 skin contralateral to ION-CCI did not affect mechanical sensitivity in either the ipsilateral side or the contralateral side, which supports that the effects of capsaicin are mediated by mechanisms localized to the ipsilateral skin (Fig. 4*A*). In our model, after capsaicin injection, mechanical threshold was not affected at 6 h, whereas it was significantly attenuated at 1 d in mice with ION-CCI (Fig. 4*B*). In mice with sham surgery, capsaicin injection produced a modest decrease in mechanical threshold at 6 h, which was not statistically significant (*p* = 0.16). In the clinic, capsaicin-induced analgesia is not permanent. Likewise, capsaicin-induced analgesia for mechanical hyperalgesia in the setting of ION-CCI was extinguished by approximately seven weeks (Fig. 4*C*). Following complete reacquisition of mechanical hyperalgesia, a second administration of capsaicin again produced similar analgesia (Fig. 4*C*, second Cap).

**Figure 5.**
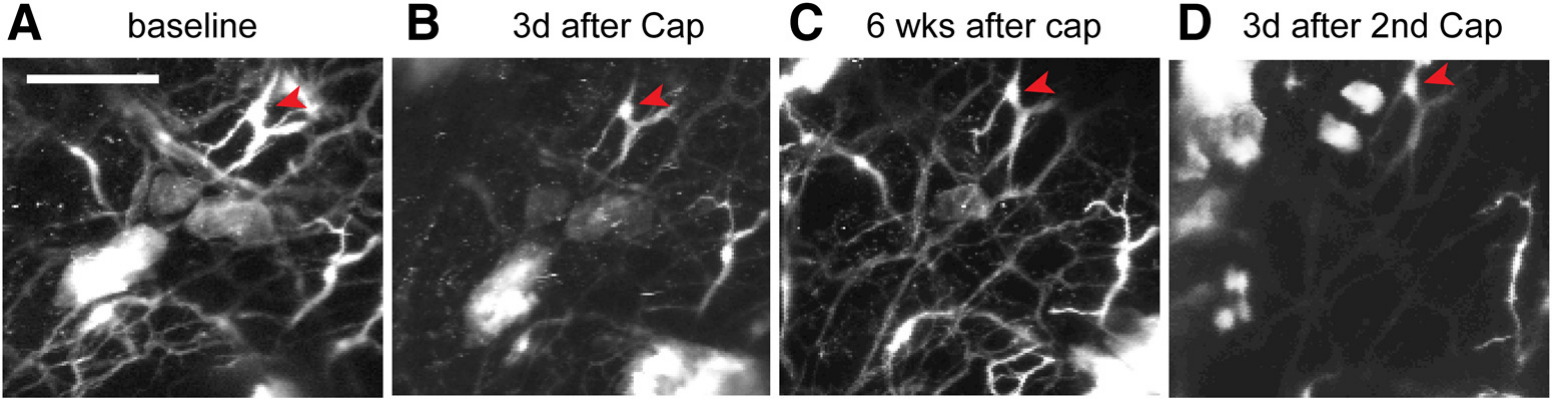
Capsaicin-induced ablation of TRPV1-LN afferent terminals is reversible and repeatable. Longitudinal monitoring of tdTomato+ afferent terminals in intact hindpaw skin of a mouse expressing tdTomato from the locus of TRPV1 (TRPV1^tdTomato^) using two-photon microscopy. To monitor capsaicin-mediated changes of tdTomato+ afferent terminals, the same site was imaged repeatedly before (***A***) and 3 d after first intraplantar injection of capsaicin (10 µg; ***B***). Six weeks after first injection of capsaicin (***C***), the fibers were regenerated. Second injection of capsaicin reproduced ablation of the fibers (***D***). Projected image stacks across ∼680 µm (340 *z*-axis sections at 2 µm). Scale bar: 100 µm.

To evaluate reversible and repeatable ablation of cutaneous afferents, we performed longitudinal *in vivo* two-photon microscopy imaging to monitor TRPV1-LN nerve terminals in intact hindpaw skin of a TRPV1^tdTomato^ mouse (Fig. 5). We longitudinally acquired images from the same skin site on multiple days. We found that intraplantar capsaicin treatment caused a reduction in the number of tdTomato+ nerve terminals after 3 d (Fig. 5*A*,*B*). Ablation occurred in a majority of tdTomato+ fibers, but some fibers were resistant to capsaicin (Fig. 5, arrowheads). This is likely due to the lack of TRPV1 expression in a subpopulation of TRPV1-LN neurons (Cavanaugh et al., 2011). The unaffected terminals served as a reference to facilitate longitudinal monitoring of the same site. When we checked the same site six weeks following capsaicin injection, the number of tdTomato+ fibers had recovered to a value similar to that at baseline (Fig. 5*C*), which was consistent with our findings in an immunohistochemical assay (Wang et al., 2017b). A second intraplantar injection of capsaicin produced ablation again (Fig. 5*D*). Overall, these results support that attenuation of mechanical hyperalgesia by capsaicin is correlated with ablation of afferent terminals in skin and that such ablation and attenuation can be conducted more than once.

### Capsaicin-induced ablation of TRPV1+ axonal terminals is necessary for capsaicin-induced analgesia

**Figure 6.**
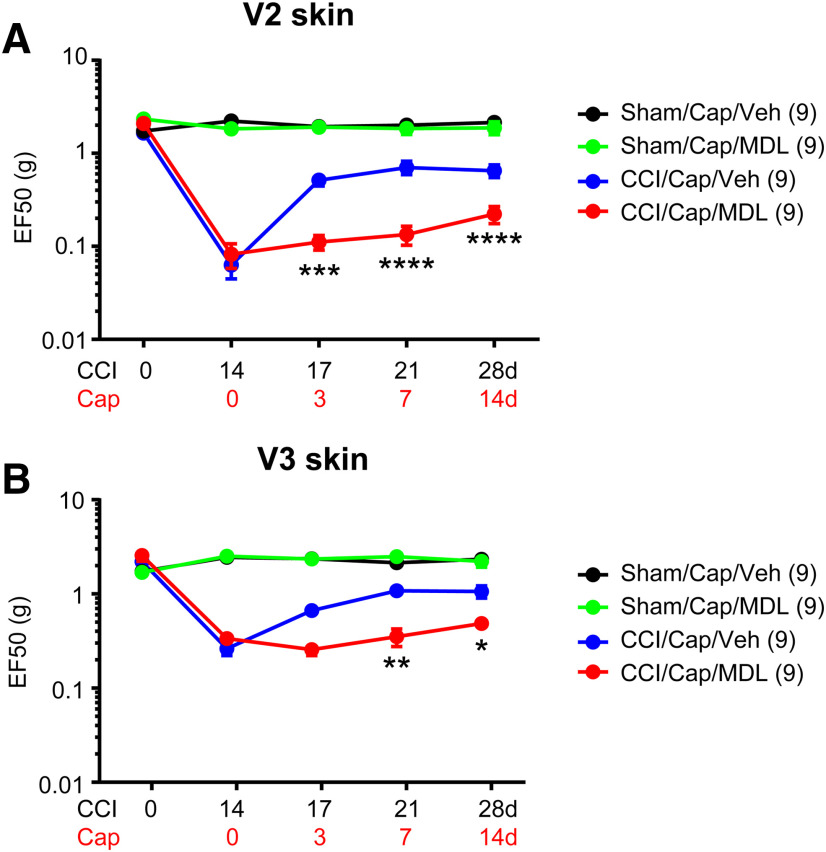
Inhibition of calpain attenuates capsaicin-induced analgesia for TNP. Mechanical hyperalgesia in V2 (***A***) or V3 (***B***) skin following ION-CCI was attenuated by injection of capsaicin (Cap; 10 µg; blue). Co-administration of capsaicin with calpain inhibitor MDL28170 (MD; 10 μm in 20 µl) into whisker pad area abolished capsaicin-induced analgesia for TNP compared with the injection of vehicle (Veh). Two-way RM ANOVA followed by Bonferroni *post hoc* test (CCI/Cap/Veh vs CCI/Cap/MDL); *p < 0.05, **p < 0.01, ***p < 0.0005, ****p < 0.0001.

**Figure 7.**
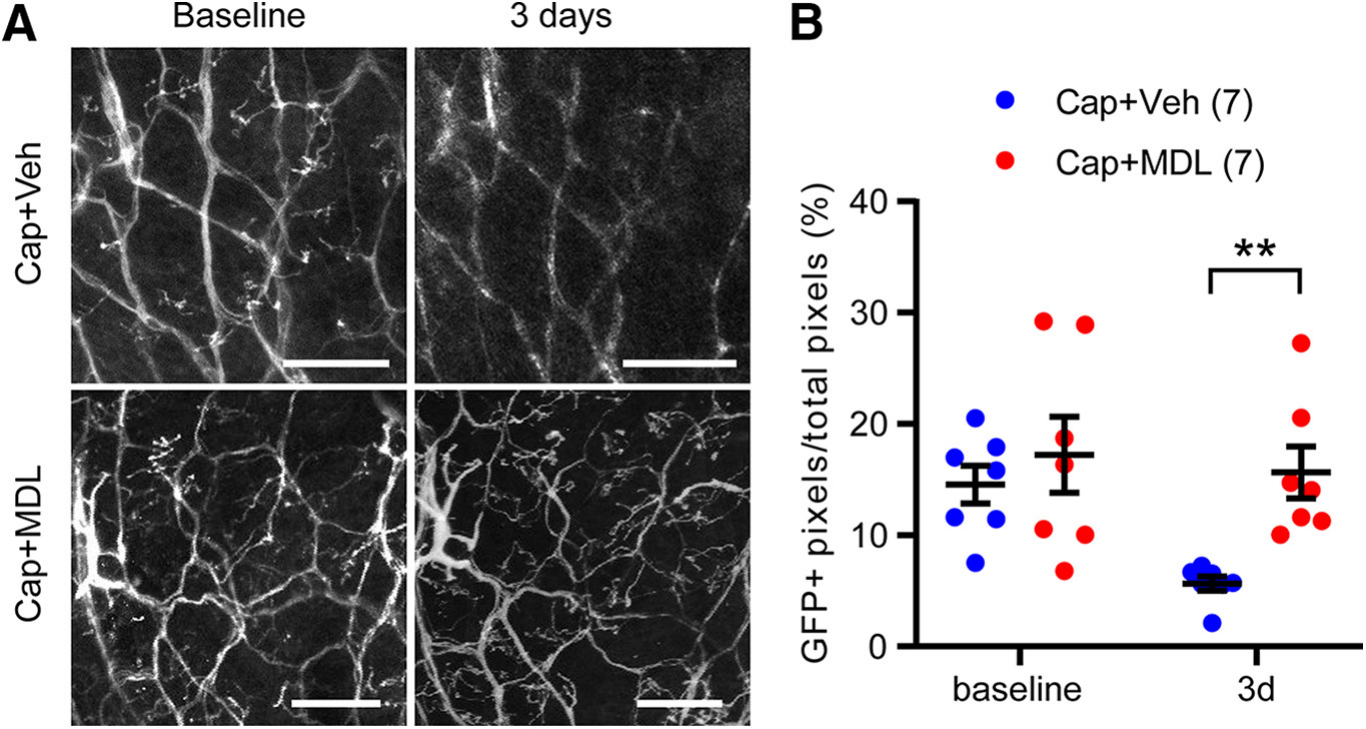
Inhibition of calpain attenuates capsaicin-induced ablation of TRPV1-LN afferent terminals in hindpaw. ***A***, Longitudinal *in vivo* two-photon imaging of GFP+ afferent terminals in intact hindpaw of a mouse expressing mGFP from the locus of TRPV1 (TRPV1^mGFP^). To assess changes of GFP+ afferent terminals, the same site was imaged repeatedly before (baseline) and 3 d after intraplantar injection of vehicle or MDL28170 (10 μm in 10 µl) followed by capsaicin (10 µg). The images represent projected image stacks across 100–150 µm (200–300 *z*-axis sections at 0.5 µm). Scale bar: 50 µm. ***B***, In each stacked image, the proportion of GFP+ pixels/total pixels in the imaged area was calculated. *N* = 7 hindpaws from 5 mice per group; **p < 0.01 in Sidak *post hoc* assay following two-way ANOVA.

To determine the role of the Ca^2+^/calpain pathway in capsaicin-induced analgesia, we tested the effects of calpain inhibition on capsaicin-induced analgesia by co-administration of MDL28170 and capsaicin. Administration of capsaicin to V2 skin robustly attenuated mechanical hyperalgesia both in V2 and V3 in mice with ION-CCI but not in sham mice (Fig. 6*A*,*B*), which is consistent with the results in Figure 2. In contrast, co-administration of capsaicin with MDL28170 did not increase mechanical threshold and substantially prevented capsaicin-induced analgesia both in V2 and V3 (interaction, *F*_(3,48)_ = 14.11, *p* < 0.0001; two-way RM ANOVA). The inhibitory effects of MDL28170 on capsaicin-induced ablation of afferent terminals were assessed through longitudinal imaging of cutaneous afferent terminals in hindpaw of TRPV1^mGFP^ mice (Fig. 7). Using two-photon microscopy, GFP+ nerve terminals were captured from identical sites before and 3 d after the injection of capsaicin+vehicle (Cap+Veh) or capsaicin+MDL28170 (Cap+MDL) to the hindpaw (Fig. 7*A*). For quantification, the proportion of GFP+ pixels in entire ROI was calculated in each field of view (Fig. 7*B*). In baseline before injection, ∼15% of area was GFP+ in both groups. Compared with the TRPV1^tdTomato^ mice (Fig. 5), TRPV1^mGFP^ mice exhibited networks of fine cutaneous afferent terminals more clearly. In 10 mice, we injected Cap+Veh or Cap+MDL to both hindpaws of five mice in each group. Three days after injection, we identified the same site by using the pattern of unaffected GFP+ afferents as a reference. We were not able to locate the identical site in three hindpaws in each group, which were excluded from the analysis. In the Cap+Veh group, overall GFP+ signals were substantially reduced; fine GFP+ fibers disappeared and the brightness of GFP in large bundles of fibers were reduced, both of which were interpreted as consequence of the ablation by capsaicin. By contrast, GFP+ signals in the Cap+MDL group were comparable to those in baseline. When we quantified the GFP signals by measuring the proportion of GFP+ pixels, the Cap+Veh group and the Cap+MDL showed significant different changes from baseline (interaction, *F*_(1,12)_ = 9.19, *p* = 0.01; two-way RM ANOVA). In *post hoc* analysis, the Cap+Veh group showed significantly less GFP+ pixels than Cap+MDL, indicating protective role of MDL28170 for capsaicin-induced ablation. These results suggest that TRPV1/Ca^2+^/calpain-dependent signaling plays a dominant role in capsaicin-induced analgesia for TNP and that capsaicin-induced ablation of afferent terminals is necessary for capsaicin-induced analgesia.

## Discussion

Capsaicin has been widely used as a tool for reliably producing pain in both clinical and preclinical studies, which is mediated by the activation of TRPV1 in nociceptive afferent terminals (Caterina et al., 2000). Although capsaicin also has long been used as a therapeutic tool to attenuate chronic pain conditions (Chung and Campbell, 2016), mechanisms underlying capsaicin-induced analgesia has been enigmatic for centuries. In this study, we showed that a single focal injection of capsaicin induces long-lasting analgesic effects on both mechanical hyperalgesia and pain aversion in ION-CCI, a clinically relevant neuropathic pain model. We also showed that capsaicin-induced ablation of TRPV1+ nociceptor terminals is closely correlated with capsaicin-induced analgesia: Time course of initiation of capsaicin-induced analgesia (1 d) is correlated with the time course of achieving full ablation of TRPV1+ afferents terminals after capsaicin injection (Wang et al., 2017b). Time course of extinction of capsaicin-induced analgesia (approximately seven weeks) is also correlated with the time required for epidermal nerve fiber regeneration following ablation (Wang et al., 2017b). A second application of capsaicin also produced ablation of nerve terminals and analgesia, which is reminiscent of the analgesia achieved by repeated application of topical capsaicin as is done in the clinic (Mou et al., 2013). Furthermore, capsaicin-induced analgesia was effectively abolished by co-administration of capsaicin and MDL28170. Based on the effects of MDL28170 preventing capsaicin-induced ablation of afferent terminals (Fig. 7; Wang et al., 2017b), we concluded that the effects of MDL28170 on capsaicin-induced analgesia are likely mediated by the prevention of capsaicin-induced ablation of TRPV1+ afferent terminals. Because of the technical difficulty of quantifying afferent terminals in hairy skin, we were not able to directly determine the association of capsaicin-induced ablation of afferent terminals in facial skin with analgesia. Despite this limitation, our data strongly support our hypothesis that capsaicin-induced ablation of peripheral nociceptive fibers is necessary for capsaicin-induced analgesia.

Capsaicin administration can cause desensitization of TRPV1 (Joseph et al., 2013), inhibition of nociceptor firing (Ma et al., 2015), or decrease in mechanotransduction (Borbiro et al., 2015). Such early effects of capsaicin on the function of primary afferents might potentially contribute to analgesic effects immediately after capsaicin injection. However, we do not think these early events contributed to the long-lasting analgesia observed in this study, since capsaicin-induced analgesia was evident at 1 d after injection but not at 6 h. Capsaicin administration could also produce analgesia through diffuse noxious inhibitory control (DNIC; Da Silva et al., 2018). It is unlikely, however, that such DNIC effects contribute to the long-lasting analgesia, since the time course of DNIC is transient (∼30 min; Da Silva et al., 2018) and capsaicin-induced DNIC is lost following neuropathic injury (Phelps et al., 2019).

The amount of capsaicin used in this study is <1% of the dose required for systemic ablation of afferents (Jancsó et al., 1985). Consistently, injection of capsaicin to the contralateral skin did not produce analgesia on the ipsilateral side, providing evidence that capsaicin exerts its effects locally. Although the extent of ablation of afferent soma in sensory ganglia following subcutaneous injection of capsaicin has not been precisely determined, the proportions of TRPV1-LN neurons in sensory ganglia were not affected by intraplantar capsaicin injection (Wang et al., 2017b), which is also consistent with localized effects (Karai et al., 2004; Yu and Premkumar, 2015). Along with the correlation between capsaicin-induced hypoalgesia and the ablation of afferent terminals in humans (Simone et al., 1998; Polydefkis et al., 2004; Ragé et al., 2010), our results suggest that capsaicin-induced localized ablation of TRPV1+ terminals is the primary determinant of capsaicin-induced analgesia.

TRPV1+ afferents and TRPV1-LN afferents do not function as mechanosensitive neurons under physiological conditions: ablation of TRPV1-LN neurons in mice eliminates heat and cold responses, but basal mechanical sensitivity is not affected (Mishra et al., 2011). Chemical ablation of TRPV1+ afferents does not affect mechanical sensitivity (Karai et al., 2004). Our previous study in the TRPV1^hM4Di^ mice also indicated that basal thermal and mechanical nociceptive threshold as well as the development of inflammatory pain were unaffected compared with TRPV1^Cre^ control mice (Wang et al., 2017a). Consistently, mechanical sensitivity in sham animals were not affected by chemogenetic silencing of TRPV1-LN afferents by CNO. However, chemogenetic inhibition of TRPV1-LN afferent terminals in mice with ION-CCI attenuated mechanical hyperalgesia, which further support the necessity of TRPV1+ afferent terminals in mechanical hyperalgesia under pathologic conditions. Of note, since TRPV1-LN afferents contain TRPV1-negative afferents (Cavanaugh et al., 2011) and TRPV1-negative TRPV1-LN neurons are mostly non-peptidergic Aδ or c-nociceptors (Patil et al., 2018), we cannot exclude the possibility that TRPV1-negative nociceptors could have contributed in the chemogenetic study. Further investigation of neuroplasticity of trigeminal afferents in TRPV1-LN, including non-peptidergic nociceptors, is warranted.

How do TRPV1+ afferents contribute to mechanical hyperalgesia after ION-CCI? Peripheral neuropathy is known to produce a wide variety of changes in TRPV1 expression in sensory ganglia (Hudson et al., 2001; Fukuoka et al., 2002; Kanai et al., 2005; Biggs et al., 2007; Christoph et al., 2007; Kim et al., 2008; Vilceanu et al., 2010; Urano et al., 2012; Hirai et al., 2014). Sensitization of TRPV1 in central terminals within the Vc contributes to synaptic facilitation of primary afferent excitatory input and maintains mechanical hyperalgesia following ION-CCI in mice (Kim et al., 2014). ION-CCI enhances overall capsaicin responses of TG neurons including some larger diameter TG neurons (Kim et al., 2014). These functional changes are consistent with the TRPV1 upregulation in larger diameter myelinated afferents reported in other trigeminal neuropathy models (Kim et al., 2008; Urano et al., 2012; Zakir et al., 2012). Peripheral nerve axotomy also induces *de novo* expression of TRPV1 in isolectin B4+ afferents (Vilceanu et al., 2010; Wang et al., 2011), a subset of mechanosensitive afferents (Cavanaugh et al., 2009). Therefore, ION-CCI possibly induces *de novo* expression of TRPV1 in mechanosensitive afferents, e.g., IB4+ afferents, which could facilitate synaptic transmission of mechanical afferent information in the central terminals to maintain mechanical hyperalgesia. Certainly, our data (Fig. 1*C*) suggest that TRPV1 molecule itself at the peripheral terminals is not responsible for transducing mechanical pain in ION-CCI. However, *de novo* expression of TRPV1 in mechanosensitive afferents after ION-CCI should render sensitivity to administered capsaicin to the peripheral terminals of these afferents, and focal capsaicin could thereby produce ablation and concomitant analgesia for mechanical hyperalgesia under ION-CCI. Any such causal contributions of neuroplastic changes of TRPV1 in distinct subsets of trigeminal afferents to hyperalgesia following ION-CCI need to be determined in future studies.

It is highly likely that capsaicin-induced ablation of nerve terminals is necessary but not sufficient to explain the full spectrum of analgesic effects of this compound and that these effects also involve supraspinal mechanisms. For example, since capsaicin-induced analgesia occurs in extraterritorial V3 skin and the secondary mechanical hyperalgesia in V3 is driven by central terminal TRPV1 sensitization (Kim et al., 2014) and descending facilitation from the rostral ventromedial medulla (Okubo et al., 2013), peripheral capsaicin injection may produce analgesia over a broad orofacial area through the normalization of descending pain facilitation after long-term attenuation of nociceptive input from injured nerves. In addition to analgesia to evoked pain, capsaicin injection also attenuates aversive features of neuropathic pain as assessed by CPP. This suggests the effects of capsaicin-induced ablation on brain structures involved in affective pain. We do not anticipate direct actions of capsaicin on the brain. Rather, capsaicin-induced ablation of nociceptive afferent terminals and consequent prolonged reduction of nociceptive inputs into the brain likely result in altered pain processing, e.g., reduced central sensitization, in regions subserving sensory-discriminative and affective components of pain. Indeed, lidocaine patch treatment attenuates spontaneous pain from postherpetic neuralgia, which is accompanied by attenuation of increased activity of pain-related brain regions (Geha et al., 2007). It will be necessary to define the plastic changes in sensory and affective pain pathways associated with capsaicin-induced analgesia in the future.

Given the paucity of data supporting the contribution of TRPV1+ afferents to hyperalgesia in spinal neuropathic pain models (Ossipov et al., 1999; King et al., 2011; Mishra et al., 2011; Abooj et al., 2016), our results in the ION-CCI model are surprising. Diverse pathophysiological mechanisms among the variety of commonly used peripheral neuropathy models might contribute to the discrepancy. Presumably TRPV1+ afferents could contribute to mechanical hyperalgesia in cases when nociceptors are relatively intact and abnormally sensitized. In our model, we used chromic gut for loose ligation of ION. Although this model does not involve substantial deafferentation, it is accompanied by strong inflammatory components due to chromic gut (Ma et al., 2012) and likely involves a greater role of TRPV1+ nociceptors and, hence, a greater degree of capsaicin-induced analgesia. It is possible that ION-CCI mimics neuropathic pain in a certain population of patients who are more susceptible to topical capsaicin treatment. Indeed, capsaicin is not effective in every neuropathic pain patient but is only effective in less than half of patients treated (Backonja et al., 2008). Recent studies have shown that patients with neuropathic pain syndromes exhibit a wide spectrum of sensory symptoms (loss or gain in thermal and mechanical sensitivity), suggesting the involvement of diverse underlying central and peripheral mechanisms (Maier et al., 2010; Baron et al., 2017). In a study of ∼900 peripheral neuropathy patients with diverse etiologies, unbiased clustering based on their sensory profiles revealed that 33% of patients formed a group showing significant sensory gain (without sensory loss; Baron et al., 2017). This group of patients showed heat and cold hyperalgesia as well as mechanical hyperalgesia (enhanced pressure and pinprick response; Baron et al., 2017). Such hyperalgesia may be mediated by intact but abnormally hyperactive primary afferent nociceptors (“irritable nociceptors”; Fields et al., 1998). These findings suggest that selectively targeting “irritable” nociceptors using capsaicin might improve pain management in this group of patients. Considering the fact that topical capsaicin was shown to be effective in alleviating TNP in humans (Epstein and Marcoe, 1994; Pastre et al., 2008; Wagner et al., 2013), our data using the ION-CCI model likely suggest that TRPV1 or TRPV1+ afferents are effective targets for treating TNP.

In conclusion, we showed that local chemogenetic silencing of peripheral TRPV1-LN afferents reduces mechanical hyperalgesia after trigeminal nerve injury. Strikingly, we further found that a single focal injection of capsaicin induces long-lasting analgesia. Capsaicin-induced analgesia is closely correlated with capsaicin-induced ablation of TRPV1+ nociceptor fibers, which supports our hypothesis that TRPV1+ afferents contribute to the maintenance of TNP and capsaicin-induced ablation of peripheral nociceptive fibers is necessary for capsaicin-induced analgesia. These results strongly suggest that TRPV1+ afferents contribute to the maintenance of TNP and their targeting could be therapeutic for treating TNP in the clinic. Further elucidation of the mechanisms underlying capsaicin-induced analgesia should facilitate improve capsaicin therapy by reducing the associated procedural pain and enhancing therapeutic efficacy.

## Synthesis

Reviewing Editor: Karen Davis, Krembil Research Institute and University of Toronto Decisions are customarily a result of the Reviewing Editor and the peer reviewers coming together and discussing their recommendations until a consensus is reached. When revisions are invited, a fact-based synthesis statement explaining their decision and outlining what is needed to prepare a revision will be listed below. The following reviewer(s) agreed to reveal their identity: Felix Viana, John Neubert. Note: If this manuscript was transferred from JNeurosci and a decision was made to accept the manuscript without peer review, a brief statement to this effect will instead be what is listed below.

The authors present the paper titled “Ablation of TRPV1+ afferent terminals by capsaicin mediates long-lasting analgesia for trigeminal neuropathic pain.” This is a well-written paper that provides a mechanism for long-lasting capsaicin-induced analgesia in a trigeminal orofacial neuropathic model in mice. The studies are designed well and the authors use the data to synthesize a mechanism of action regarding the capsaicin effects on mechanical sensitivity in a mouse chronic nerve constriction injury. The data and interpretation is straight-forward and the Discussion is appropriate. The authors have addressed criticisms raised in my previous review, performing additional experiments and rewriting some sections of the manuscript. I think they have produced a very fine study. There remains some minor issues to address:

1. INTRODUCTION: Could use more development, especially in terms of the description/rationale for calpain-induced ablation of TRPV1+ fibers (pages 4 and 5).

2. METHODS:

- Intra-Vc microinjection of drug - no details of what drugs were injected here. Could piece together with the description in the Results, but would be appropriate to put in Methods to prepare the reader.

- Details of the capsaicin injection are important - was it injected as a single bolus or peppered within the V2 distribution?

- How long after capsaicin injection were animals tested with von Frey

- It is unclear the amount of “spread” of the injection in terms of lesioning effects. There is likely a gradient dilution effect in the tissue, with lesioning directly at the bolus site, but possible partial effects distant to this site. This could possibly lead to increased sensitivity due to fewer TRPV1+ fibers being ablated or even an increase of fibers being activated by this diluted dose at the edge of the injection site.

3. RESULTS:

- Much of the write-up in the Results seems like it should be in the Introduction or Discussion. It is rather confusing to try to figure out what the results were from this paper versus previous work.

- This description in Fig. 2 can go in Fig. 1: “Mechanical sensitivity is presented as EF50...represent the number of mice in each group.”

- Figure 3C is redundant with 3B - consider removing

- Fig. 4A figure legend is still a bit confusing with the use of contralateral to describe the side of injection and the opposite side of the CCI - try to clarify.

- Fig. 4B - would be interesting to see the Sham results here to evaluate capsaicin-induced hyperalgesia/allodynia at this 6 hr time point

4. DISCUSSION:

- Use of capsaicin is an established tool for producing pain in both clinical and preclinical models in order to study pain mechanisms or evaluate pain treatments.

- The statement presented in the initial paragraph of the discussion is arguably not fully correct. The authors state “capsaicin-induced ablation of TRPV1+ nociceptor terminals localized to the ipsilateral skin contributes to its analgesic effects on both mechanical hyperalgesia and pain aversion in a clinically relevant neuropathic pain model.” In my opinion, the authors clearly show that capsaicin causes ablation of cutaneous terminals, but they obtained these results in the hairless skin of a the hindpaw, and in the absence of any neuropathic condition!!, a different experimental condition and innervation territory than the one used for examining the behavioral changes. I feel they should rephrase the statement to more accurately reflect their findings. A similar caution should be introduced in drafting the last paragraph. In other words, the explanation provided to explain the results (ablation of TRPV1+ terminals) is very likely, but it was never tested in the peripheral territory used for the behavioral evaluation.
